# Acute Infarction Due to Extrinsic Coronary Compression by Left Ventricular Pseudoaneurysm Following Endocarditis Surgery—Case Management

**DOI:** 10.1093/icvts/ivag115

**Published:** 2026-04-22

**Authors:** Lars Niclauss, Mario Verdugo, Matthaios Papadimitrou Olivgeris, Kirsch Matthias

**Affiliations:** Department of Cardiovascular Surgery, University Hospital of Lausanne (CHUV), Lausanne CH-1011, Switzerland; Department of Cardiovascular Surgery, University Hospital of Lausanne (CHUV), Lausanne CH-1011, Switzerland; Department of Infectious Diseases and Infection Prevention, University Hospital of Lausanne (CHUV), Lausanne CH-1011, Switzerland; Department of Cardiovascular Surgery, University Hospital of Lausanne (CHUV), Lausanne CH-1011, Switzerland

**Keywords:** left main coronary artery compression, acute myocardial infarction, endocarditis surgery, myocardial perfusion imaging, aortic root reconstruction, Rubidium-82 positron emission tomography computed tomography

## Abstract

Extrinsic coronary compression is rare, difficult to diagnose, and can have serious consequences. A patient who had surgery for aortic valve endocarditis with patch closure of a septal abscess was readmitted for myocardial infarction. CT scan revealed compression of the left main stem due to pseudoaneurysm formation under the patch. Positron emission tomography confirmed acute hypoperfusion of the left ventricle, making immediate high-risk reoperation unavoidable. Imaging of the patient at one-year follow-up showed complete thrombosis of the former abscess cavity. Extrinsic coronary compression due to an abscess/pseudoaneurysm is associated with a high mortality rate and surgery appears to be the only possible life-saving treatment. In cases of unclear cardiac symptoms occurring under these circumstances (and thus suggesting extrinsic coronary compression), early imaging for quantitative myocardial perfusion should be considered to assess severity of myocardial ischaemia and thus urgency of surgery.

## INTRODUCTION

Extrinsic coronary compression is rare but potentially life-threatening. The most frequently described aetiology is a compression due to dilated pulmonary arteries in pulmonary hypertension (PTH).^1^ Galié et al. found significant stenosis of the common trunk in 30% of all PTH patients with coronary symptoms.^2^ Compression by a pseudoaneurysm is much rarer.

## CASE PRESENTATION

A man in his late fifties, successfully treated for high-grade papillary urothelial carcinoma with Pembrolizumab via a subclavian port-a-catheter, developed septic thrombosis with circulating methicillin-sensitive *Staphylococcus epidermidis*. Six days after catheter removal, the patient presented with cardiogenic shock caused by severe aortic regurgitation due to native valve endocarditis. Coronary angiography revealed no stenotic lesions and normal coronary anatomy.

The aortic valve was replaced with a biological prosthesis (Inspiris Resilia). Intraoperatively, a non-perforating abscess cavity in the muscular septum, approximately 2-3 cm in diameter, was reconstructed with a xeno-pericardial patch. The postoperative course was favourable; an Angio-CT scan on day 3 showed a small residual pseudoaneurysm of the reconstructed left ventricular outflow tract (LVOT) without contrast leakage, and the patient was discharged while continuing outpatient intravenous flucloxacillin therapy.

**Figure 1.**
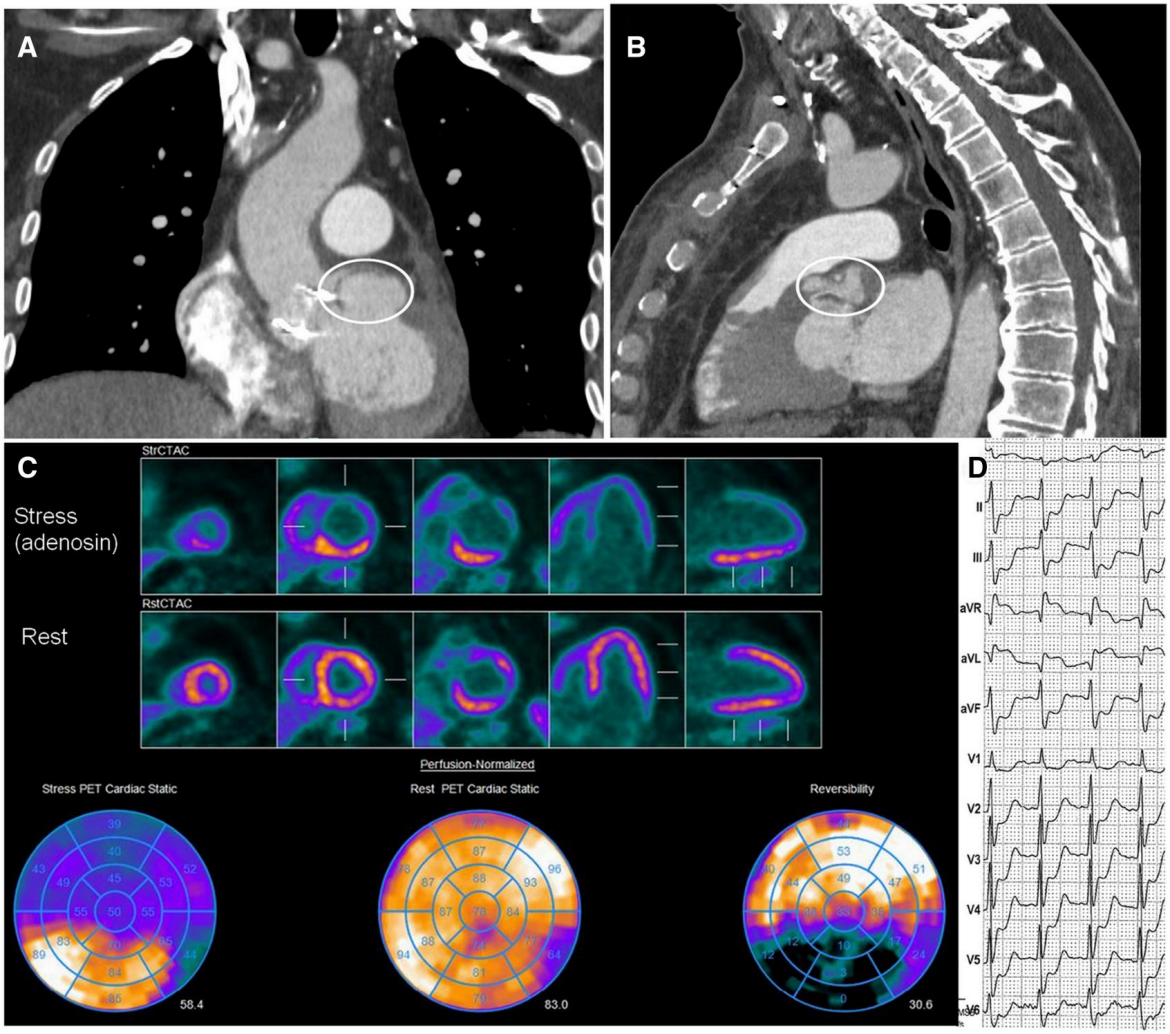
Coronal A) and Sagittal Plan B) of the Angio-CT Showing LVOT Pseudoaneurysm. Stress ischemia during C) ^82^Rb-PET/CT with D) significant ECG modifications

On the 25th day after surgery, the patient, who was still receiving antibiotics, was readmitted due to acute chest pain accompanied by an increase in high-sensitivity troponin T to 716 ng/l (upper limit of the reference interval 14 ng/l). Angio-CT scan now revealed a marked enlargement of the LVOT pseudoaneurysm, which lifted the left main stem and pushed it under the pulmonary artery (circle in **Figure 1A and B**). The clinical symptoms resolved spontaneously and rapidly, and the subsequent troponin levels—581 ng/l (after 6 hours) and 515 ng/l (after 12 hours)—initially suggested progressive normalization, with the patient remaining asymptomatic. This initial decline in troponin then plateaued and rose slightly to 533 ng/l after 36 hours, so the patient, who was still asymptomatic at that time, underwent rubidium-82 positron emission tomography/computed tomography (^82^Rb-PET/CT). This showed, under adenosine, stress-induced severe LV ischaemia, diffuse ST-segment depression and aVR ST-segment elevation on ECG, confirming functional, severe stenosis of the left main stem (**Figure 1C and D**). In addition, the patient experienced a recurrence of chest pain during the stress test, making urgent reoperation unavoidable.

**Figure 2.**
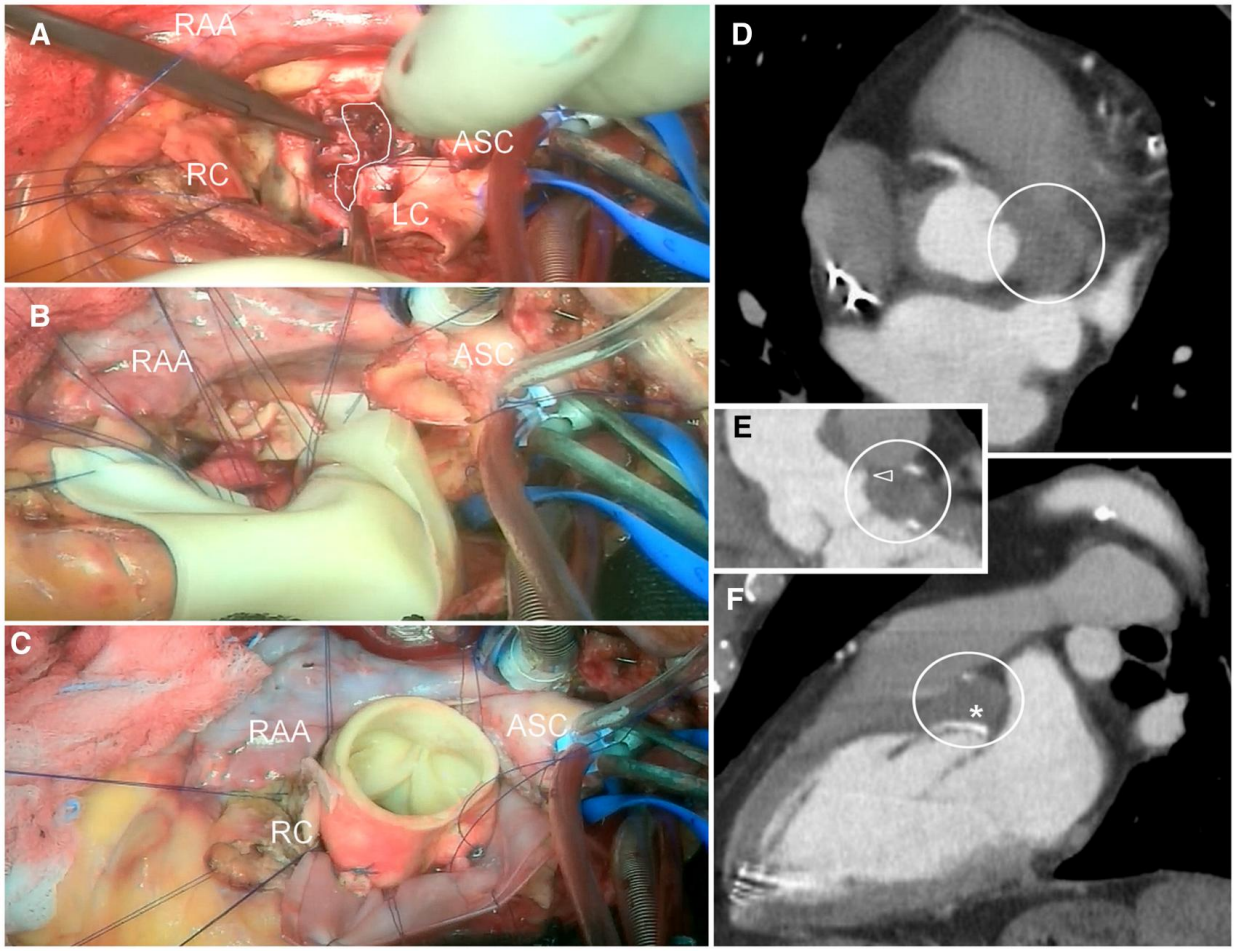
(A-C) Intraoperative View. (D-F) Angio-CT after 1 Year (Asterisk: xeno-Pericardial Patch in the LVOT; Triangular Arrow: Sub-Occlusion of the Left Main Stem).

After complete resection of the aortic root and mobilization of the coronary ostia, the pseudoaneurysm was incised directly from the cranial side *(white boundary line in* **Figure 2A**). Although, as shown in **Figure 2A**, the left coronary ostium appeared intact, the left main stem was partially eroded and could not be reimplanted. The former abscess cavity was significantly dilated, likely due to partial dehiscence of the proximal suture, and drained spontaneously into the LVOT. There were no obvious signs of acute reinfection (pus, vegetations, etc.), but the tissue at the margin of the cavity was very friable, edematous, and not firm. Consequently, the LVOT and the entire aortic annulus were reconstructed using a folded xeno-pericardial patch placed as far as possible into the LV, near the base of the papillary muscles (**Figure 2B**).

The aortic root was replaced with a biological conduit (Medtronic Freestyle 25 mm for Bentall procedure; **Figure 2C**). The second side of the folded patch was used to isolate the exposed (no longer perfused) abscess cavity from the adjacent cardiac structures. Both left main coronary arteries were bypassed using venous grafts and weaning from the heart-lung-machine was achieved under aminergic support. Transoesophageal echo showed mild left ventricular dysfunction (LV ejection fraction of 45%) and no paravalvular leakage (mean/maximal transvalvular gradient of 8 and 20 mmHg). Following progressive recovery, the patient was discharged four weeks later.

The follow-up after 2 months showed a small, persistent pseudoaneurysm (17 × 11 mm), which was completely thrombosed after 1 year (*circle in* **Figure 2D-F**).

## DISCUSSION

In their review, Joy et al. describe 12 patients with acute coronary syndrome (ACS) caused by direct coronary compression due to an aortic root abscess.^3^ Eight of these (67%) underwent surgery, of whom 6 survived, corresponding to a perioperative mortality of 25%. The remaining 4 patients died in hospital (2 before the decision to operate, 1 who refused surgery, and 1 who had coronary stent implantations), resulting in an overall mortality of 50%.^3^ Thus, the chances of survival appear to be higher with complex surgical repair than with conservative treatment alone.

Gharakhanian et al. recently described a patient who—similar to the present case—complained of chest pain and shortness of breath 1 month after a previous aortic root reconstruction but showed no signs of myocardial ischaemia.^4^ The diagnosis was delayed until coronary CT angiography revealed *“100% compressive occlusion of the left main artery … during systole …”*.^4^ At the time of successful reoperation, the patient had already suffered an irreversible myocardial infarction with impairment of his ventricular function.^4^

Early, targeted diagnosis of suspected extrinsic coronary compression as a possible postoperative complication through direct quantitative assessment of myocardial perfusion (as in the present case using 82Rb-PET/CT) therefore appears essential.

## Data Availability

The data underlying this article are available in the article.
